# Fear inoculation among snake experts

**DOI:** 10.1186/s12888-021-03553-z

**Published:** 2021-10-30

**Authors:** Carlos M. Coelho, Jakub Polák, Panrapee Suttiwan, Andras N. Zsido

**Affiliations:** 1 Faculty of Psychology, Chulalongkorn University, Bangkok, Thailand; 2 University Institute of Maia, Maia, Portugal; 3 Center for Psychology at University of Porto, Porto, Portugal; 4 Applied Neuroscience and Neuroimaging Research Programme, National Institute of Mental Health, Klecany, Czech Republic; 5 Department of Psychology, Faculty of Arts, Charles University, Prague, Czech Republic; 6 Life Di Center, Faculty of Psychology, Chulalongkorn University, Bangkok, Thailand; 7 Institute of Psychology, University of Pécs, Pécs, Hungary

**Keywords:** Fear of snakes, Fear immunization, Hypophobia, Preparedness theory, Snakebite, Snake questionnaire

## Abstract

**Background:**

Fear acquisition of certain stimuli, such as snakes, is thought to be rapid, resistant to extinction, and easily transferable onto other similar objects. It has been hypothesized that due to increased survival chances, preparedness to instantly acquire fear towards evolutionary threats has been hardwired into neural pathways of the primate brain. Here, we compare participants’ fear of snakes according to experience; from those who often deal with snakes and even suffer snakebites to those unfamiliar with snakes.

**Methods:**

The Snake Questionnaire-12 (SNAQ-12) and Specific Phobia Questionnaire (SPQ) were administered to three groups of participants with a different level of experience with snakes and snakebites: 1) snake experts, 2) firefighters, and 3) college students.

**Results:**

This study shows that individuals more experienced with snakes demonstrate lower fear. Moreover, participants who have suffered a snakebite (either venomous or not) score lower on fear of snakes (SNAQ-12), but not of all other potentially phobic stimuli (SPQ).

**Conclusions:**

Our results suggest that a harmless benign exposure might immunize people to highly biologically prepared fears of evolutionary threats, such as snakes.

## Background

It has been estimated that each year, venomous snakebites kill about 94,000 people worldwide [1, 2]. According to the World Health Organization, the annual number of fatalities could be as high as 138,000, with additional 400,000 amputations and other severe health consequences [3]. Therefore, the WHO categorized snakebite envenomation as a highly neglected tropical disease with the top priority for new antivenom research. Given the threat posed by snakes to humans, it is no wonder that these are one of the most feared animals and snake phobia ranks among anxiety disorders with the highest prevalence in the general population [4]. Several theoretical models have been proposed to explain this widespread, universal pattern of snake fear. Recent models are based on Seligman’s preparedness theory of phobias [5], which claims that fear responses are more readily acquired to stimuli that were relevant to the species survival throughout evolution, such as those associated with predators (e.g., snakes). Individuals able to learn to fear and avoid threatening animals, object, or situations increased their survival chances. Consequently, these fears gradually became genetically fixed in the form of predisposition or preparedness to be 1) rapidly acquired and 2) more resistant to extinction once developed.

Seligman’s theory triggered a large number of laboratory studies both on humans [6] and non-human species [7–9]. For example, naïve laboratory-reared macaques without any prior experience with snakes acquired an intense fear response just by observing another monkey behaving fearfully towards a snake. In contrast, observing a fearful display to a neutral object such as a flower was not effective in inducing fear of flowers [7]. More recent studies suggest that snakes are indeed a particular class of stimuli for humans (e.g., [10–14]). Snakes are detected much faster than other stimuli [15], even when attentional and visual conditions are challenging [16, 17]. The coiled snake body shape [18], striking position [19], as well as the typical diamond-shaped scales of the snakeskin [20] might be critical distinctive visual features able to preferentially attract heightened human attention. This might be the mechanism that makes snake fear easy to acquire [21].

According to Seligman’s theory, a single exposure (even an indirect one) to snakes and other evolutionary relevant threats should be sufficient for fear acquisition. However, there is also a line of evidence showing that repeated previous experience with real situations might give people a sense of control over it and thus lower their fear, possibly preventing a phobia from developing even after a future harmful experience [22]. This phenomenon could be referred to as fear immunization, latent inhibition, or fear inoculation. Fear immunization is more often related to the participant’s observation of other people’s non-fearful experiences (modeling), whereas latent inhibition is preferably associated with a simple frequent exposure or neutral familiarity with a certain stimulus [23].

In behavioral sciences, the latent inhibition refers to the retarded acquisition of a conditioned response that occurs if the tested participant was previously exposed to the to-be-conditioned stimulus without a consequence of a paired unconditioned stimulus (UCS) [24, 25]. On the neural level, the underlying mechanism of latent inhibition could be similar to extinction producing an inhibitory memory in the infralimbic cortex [26]. When subjects are exposed to a stimulus without a UCS, they might learn its ‘irrelevance’ in terms of danger, and the subsequent reduced acquisition of the CS-UCS association is thought to reflect the process of overcoming this learned irrelevance [27] or inattention [28] and stimulus familiarity [29]. For example, Mineka and Cook [30] showed that monkeys that had been immunized against fear did not acquire fear of snakes, contrary to the latent inhibition group that did. Indeed, a recent study [31] demonstrated that pre-exposure could limit social fear acquisition even in humans. Nevertheless, previous studies only showed the immunization effect of vicarious learning on fear acquisition in situations where participants observed the behavior of a model but did not examine the effects of information or instructional learning (which is a similar indirect learning path in Rachman’s theory [32]). Although the term fear inoculation is different from the latent inhibition, they have in common the idea that exposure to certain stimuli can reduce fear acquisition and might even lead to hypophobia (extremely reduced fear in dangerous situations; see also [33]). For example, people with many opportunities for a direct contact with certain stimuli, or just seeing others having a harmless contact with these, often develop less fear after an aversive encounter (e.g., snakebite) compared to people with no contact at all (or just very rare) with the same stimuli. This was demonstrated for fear of dogs [34] and dental fears [35, 36].

Although fear of snakes is one of the most common anxieties in the general population [37, 38], there is a great lack of studies examining fear inoculation towards snakes in humans with varying levels of experience with snakes, including a harmful one (snakebite). Just recently, Onyishi and colleagues [39] studied attitudes toward snakes among Igbo people in Nigeria who differed in frequency of encounters with snakes and experience with snakebites. The authors found that more frequent encounters with snakes were negatively correlated with fear and disgust of snakes, and positively correlated with tolerance attitudes. Therefore, exposure to snakes had a positive effect on attitudes and behaviour. However, having been bitten by a snake had exactly the opposite effect. In their study, a total of 10% of people reported at least one snakebite in their lifetime, and such an experience was associated with a higher probability of killing snakes.

In this study, we explored fear of snakes among people used to deal with snakes daily and who have been repeatedly bitten by a snake compared with participants unfamiliar with snakes. Based on the preparedness theory, snake fear should be rapidly acquired and show enhanced resistance to extinction or inoculation compared to other fear-relevant but non-prepared stimuli (i.e. sensitization). Despite that, continued experience with snakes might partially override such “preparedness” and resistance of snake fear, and people frequently encountering snakes would eventually become less fearful of them (i.e. habituation, although this effect should still be lower for snakes compared to other stimuli). would expect a generalization effect of reduced fear not only of snakes but other fear-relevant animals (such as worms, lizards, spiders) too [40].

Finally, due to the importance and adaptive nature of social learning [32, 41] we also explored other potential sources of fear acquisition (effects of information or instructional learning). Social learning of fear through information has similar underlying neurobiological mechanisms to associative and prepared learning [41] and might have affected human evolution [42, 43]. Thus, it was also considered in this study.

## Methods

### Participants

The study was carried out in 2019. There were three groups of participants: 1) snake experts, including veterinarians, venom extractors, or scientists (*n* = 14, 1 female, mean age = 30.9, SD = 5.53) working at the Bangkok Red Cross (Queen Saovabha Memorial Institute) used to handle venomous snakes on a daily bases, either when showing them to visitors for educational purposes or extracting venom for research and production of antivenom; 2) firemen (*n* = 28, all males, mean age = 37.1, SD = 4.49) also used to deal with snakes (mainly nonvenomous python snakes) when they have to catch and remove them from houses, particularly during the hot wet seasons; and 3) students at Chulalongkorn University, (*n* = 71, 51 females, mean age = 20.9, SD = 0.75) without any experience with handling snakes, as a control group. The required sample size for this study was determined by computing estimated statistical power for the planned tests (pairwise comparison, correlational analyses, and ANOVA) with f = .40 and β > .8. The analysis for correlation indicated the largest required total sample size of 84.

### Assessments

#### Snake questionnaire (SNAQ-12)

We applied a specific questionnaire pertaining to fear of snakes. The SNAQ-12 [44] is a 12-item scale, where participants indicate whether they agree or not with a statement, e.g., ‘*I dislike looking at pictures of snakes in a magazine’*. The SNAQ-12 has been shown to have good internal consistency with a Cronbach’s alpha of 0.89 on this sample (item-total correlations were between 0.36 and 0.78). Confirmatory factor analysis also showed that the original one-factor structure of the questionnaire was retained on this sample (χ^2^_(54)_ = 38.22, *p* = 0.95, NFI = 0.97, RFI = 0.97, RMSEA = 0, 90%CI = 0–0.01, SRMR = 0.08). The optimal balance between sensitivity and specificity of the SNAQ-12 was achieved using a cut-off score of > 7.5, which yielded a sensitivity of 0.909 and specificity of 0.905. This suggests that someone scoring ≥8 on the SNAQ-12 should be considered as potentially a snake phobic. This test has excellent discriminatory power and thus is useful as a diagnostic tool for snake phobia [44].

#### Specific phobia questionnaire (SPQ)

The SPQ [45] is a 43-item measure of fear and interference for a broad range of objects and situations that could be clustered into five subscales (Animals, Natural Environment, Situational, Blood-Injection-Injury, and Other). Participants rate their fear and fear interference with their daily lives of a given object or situation on a 5-point Likert scale from 0-No Fear/Interference to 4-Extreme Fear/Interference, respectively. The SPQ has shown good psychometric properties and validity even in an anxiety disorders sample [45]. Therefore, the SPQ is a valid measure for screening of specific phobias. We decided not to use the Other subscale as it consists only two items and has relatively low Cronbach’s alpha (0.72), Cronbach’s alpha values for the remaining four subscales were in the range of 0.88–0.93.

#### Other questions

In addition to the SNAQ-12 and SPQ, we gathered general information about the participants’ experience with snakes and other animals. First, we asked them to estimate the *number of encounters* with a live snake in the past year and if they have ever been *bitten or injured* by any snake – and whether it was venomous or not. We also asked them if they have or have not been attacked by other animals than snakes. Participants also reported whether they thought the information about animals from the media and stories of others had *influenced their fear* (with the following choice of responses: no influence, influence on fear, influence on not to fear, and having influence both on fear and on not to fear). Finally, we evaluated the participants’ previous *experience with snakes*, inquiring about their familiarity with snakes (frequency of experience). Ratings were scored from 0 (no experience) to 4 (a lot of experience).

### Statistical analysis

Since we could not match the groups regarding the age and male-female ratio, we entered these variables in the analyses where possible as independent variables to control for their effects. Because of this imbalance, we were not interested in results concerning these variables, and thus, we only report results concerning the SPQ, SNAQ, and the other questions listed in the previous section. Further, due to the non-normal distribution of our variables, robust alternatives were used. We employed the Mann-Whitney U test to examine differences between those who have been reportedly bitten by a venomous or nonvenomous snake and those who have not. We repeated the same analysis on two subgroups (reportedly encountered a snake or not) of the control group. Rank biserial correlation (r) values are reported as effect sizes for the Mann-Whitney U tests. Then, we used the Spearman correlation analysis to explore the relationship between previous experience (i.e. number of encounters and experience rating) and fear of snakes (SNAQ-12 and SPQ). We used the Mann-Whitney test to compare people bitten by other animals and those who have or have not seen others being bitten by a snake. One-way ANOVA was performed to examine the differences between people with various previous information on snakes (i.e. no influence, influence on fear, influence on not to fear, and influence on both fear and not to fear). Finally, we analyzed possible transfer effects of fear inoculation to other subtypes of specific phobia using the SPQ subscales. For these analyses, the snake item was removed from the Animal subscale. We used the Spearman correlation to assess the relationship between experience and frequency of encounters with snakes and SPQ subscales.

Furthermore, we carried out an independent moderation analysis separately for each SPQ subscale with the SPQ subscale as a dependent variable, the SNAQ-12 as a predictor, and previous experience with snakes as a moderator to separate the direct effect of snake fear on the SPQ animal subscale and the indirect effect of it through experience with snakes. One-way Kruskal-Wallis ANOVAs were used to measure group differences on the SPQ subscales. For the statistical analyses, the JAMOVI program Version 1.0 for Windows [46] was used.

## Results

Descriptive statistics showed that none of the experts (0%), two of the firemen (7.41%), and 15 students (22.39%) reached the cut-off criteria for snake phobia on the SNAQ-12. Table 1 shows the central tendencies of each group.

**Table 1:** Central tendencies (mean scores with standard deviations in parentheses) for the three groups participating in our study on Snake Questionnaire total score (SNAQ-12), Specific Phobia Questionnaire relevant item (SPQ), and previous experience

Group	SNAQ-12	SPQ	Experience
Expert group on a snake farm	0.36 (1.08)	0.21 (0.43)	4.00 (0.00)
Firemen	3.15 (3.17)	0.78 (0.85)	3.18 (0.82)
Students	4.37 (3.62)	1.73 (0.21)	0.93 (0.75)

### The effect of snakebite on fear level

People bitten by a snake (*n* = 19) (either venomous or not) scored lower on the SNAQ-12 when compared to those never bitten; mean difference = 3.54 (U_(106)_ = 279, *p* < 0.001, r = 0.67). This was confirmed by their response on the SPQ survey. Again, people bitten by a snake scored lower; mean difference = 1.16 (U_(109)_ = 363, *p* < 0.001, r = 0.57). We also found that people bitten by a snake had more experience with snakes than those who have not been bitten; mean difference = 2.43 (U_(110)_ = 115, *p* < 0.001, r = 0.87)^1^; see Table 2 for the descriptive statistics. Results remained very similar when we compared participants bitten by a venomous snake and those never bitten; the SNAQ-12 mean difference = 3.26 (U_(106)_ = 173.5, *p* = 0.002, r = 0.61); the SPQ mean difference = 1.30 (U_(109)_ = 158, *p* < 0.001, r = 0.67); and the experience mean difference = 2.31 (U_(110)_ = 81, *p* < 0.001, r = 0.83); see Table 3 for the descriptive statistics. Interestingly, the same nonsignificant trends could be observed in the control group (never bitten): Students with some experience with snakes scored lower on both the SNAQ-12 (mean difference = 1.68 (U_(65)_ = 385, *p* = 0.068, r = 0.27) and SPQ (mean difference = 0.45 (U_(68)_ = 435, *p* = 0.1, r = 0.23)). That is, experience in itself, regardless of a history of snake bite, may lower the chances of developing higher levels of fear.^2^

**Table 2:** Central tendencies for those who have and have not been bitten by a snake on the Snake Questionnaire-12 total score (SNAQ-12), Specific Phobia Questionnaire relevant item (SPQ), and previous experience

	Group	N	Mean	Median	SD
SNAQ-12	Bitten	19	0.63	0.00	1.61
	Not bitten	89	4.17	4.00	3.52
SPQ	Bitten	18	0.33	0.00	0.49
	Not bitten	93	1.49	2.00	1.21
Experience	Bitten	19	3.90	4.00	0.32
	Not bitten	93	1.46	1.00	1.21

**Table 3:** Central tendencies for those who have and have not been bitten by a venomous snake on the Snake Questionnaire total score (SNAQ-12), Specific Phobia Questionnaire relevant item (SPQ), and previous experience

	Group	N	Mean	Median	SD
SNAQ-12	Bitten	9	0.56	0.00	1.33
	Not bitten	99	3.82	3.00	3.54
SPQ	Bitten	9	0.11	0.00	0.33
	Not bitten	102	1.41	1.00	1.20
Experience	Bitten	9	4.00	4.00	0.00
	Not bitten	103	1.69	1.00	1.35

### The relationship between previous experience and fear level

Experience as well as the number of encounters with snakes correlated negatively with a moderate effect size with the SPQ snake item (rho = −0.427, *p* < 0.001 and rho = − 0.499, *p* < 0.001, respectively) and the SNAQ-12 (rho = − 0.388, *p* < 0.001 and rho = − 0.44, *p* < 0.001, respectively). People who have or have not been attacked by other animals than snakes did not differ in terms of fear of snakes (ts < 1, ps > 0.1). Furthermore, people who have seen others been bitten by other animals showed less fear of snakes; the SNAQ-12 mean difference = 1.33 (U_(106)_ = 1146, *p* = 0.057, r = 0.21); the SPQ mean difference = 0.50 (U_(109)_ = 1164, *p* = 0.027, r = 0.24) than those who have not had such an experience. This difference, however, disappeared when we excluded participants who have themselves been bitten.

### The effect of previous information on snakes on fear level

Previous information on animals had a significant effect on the SNAQ-12 (F_(3,104)_ = 5.63, *p* < 0.001, ɳ^2^_p_ = 0.14) and SPQ score (F_(3,107)_ = 4.85, *p* = 0.003, ɳ^2^_p_ = 0.12). For the SNAQ-12, Tukey-corrected follow-up pairwise comparisons showed that people in the no influence group and influence on not to fear group scored lower than those in the influence on fear group (t_(104)_ = 3.12, *p* = 0.013; t_(104)_ = 3.37, *p* = 0.006; respectively). For the SPQ, Tukey-corrected follow-up pairwise comparisons revealed that people without influence scored significantly lower than people influenced on fear (t_(107)_ = 3.38, *p* = 0.006) and people influenced both on fear and not to fear (t_(107)_ = 2.55, *p* = 0.058), but this difference was only marginally significant. The other groups did not differ significantly.

### Possible transfer effects of fear inoculation to other subtypes

Regarding the possible transfer effects of fear inoculation to other subtypes of specific phobia using the SPQ subscales, we found that frequency of encounter and experience with snakes correlated negatively with the Animal subscale with moderate effect sizes (rho = − 0.48, *p* < 0.001 and rho = − 0.43, *p* < 0.001, respectively), the Natural Environment with small effect sizes (rho = − 0.21, *p* = 0.031 and rho = − 0.200, *p* = 0.038, respectively) and Blood-Injection-Injury with small effect sizes (rho = − 0.23, *p* < 0.001 and rho = − 0.15, *p* = 0.133). The SPQ Situational subscale did not correlate with these variables (rhos < 0.1).

The three groups differed on the Animal subscale (χ^2^_(2)_ = 24.7, p < 0.001) with students scoring higher levels of fear compared to experts (W = 4.13, *p* = 0.003) and firemen (W = 6.36, p < 0.001) according to the DSCF pairwise comparisons, as well as on the Natural Environment subscale (χ^2^_(2)_ = 7.39, *p* = 0.025) where students scored higher than firemen (W = 3.55, *p* = 0.012). Similarly, the groups differed on the Blood-Injection-Injury subscale (χ^2^_(2)_ = 6.21, *p* = 0.045) with students scoring higher than snake experts (W = 3.29, *p* = 0.02). Importantly, groups did not differ on the situational subscale scores the SPQ (χ^2^_(2)_ = 1.09, *p* = 0.581). In addition, the moderation analysis revealed that the direct effect of the SNAQ-12 score on the SPQ animal subscale score was positive (0.292 to 0.666, with a point estimate of 0.479, Z = 5.02, *p* < 0.001). The effect of previous experience with snakes was negative (− 1.351 to − 0.433, with a point estimate of − 0.892, Z = 3.81, *p* < 0.001), and there was no interaction between the effects (Z = 1.68, *p* = 0.092). The effect of previous experience with snakes was nonsignificant for the Natural Environment, the Blood-Injection-Injury, and the Situational subscales.

## Discussion

This study compared participants’ fear of snakes according to experience, from people used to deal with snakes almost on a daily basis to those who are unfamiliar with them. The main findings show that more experienced individuals are at the same time less fearful of snakes than people with no experience. Even those who have been a victim of snakebite several times scored lower on fear of snakes. Regarding fear acquisition pathways, social transmission seems to play a role in learning snake fear.

Data suggest that certain people can become immunized to biologically-prepared fearful stimuli, such as snakes, despite aversive experiences, if given a certain amount of exposure and familiarity. It could be argued that these participants might be fearless and have overall low general ability to learn fear. However, high and low fear groups did not differ on the situational subscale scores, suggesting that lower scores presented on the animal subscale were not due to a general lack of fear, but more likely due to the significant amount of experience and direct contact with snakes. Our results are not completely unmatched in previous research. Nelson and colleagues [47] showed that targeting the cost of feared outcomes (i.e. purposely acting in a foolish manner during public speaking to learn that embarrassment or negative evaluated would not be as unbearable imagined) instead of the probability (i.e. practice public speaking to learn that the outcomes one fears are unlikely to happen) was more successful in terms of lowering fear levels. Similarly, a snakebite might produce a reduction in fear level, if the bite is not as costly in terms of physical harm as was anticipated.

We also noticed that participants with lower levels of snake fear showed less fear of the Natural Environmental and Blood-Injury-Injection subtypes as well as other animals. This might be due to transfer effects between different subtypes of specific phobias. The acquired sense of control over snakes might cross over to the whole category (i.e. animals). Generalization between different subtype categories might also result from the experience with the snakes’ natural environments (e.g., deep water, enclosed spaces, swimming, open spaces) and injuries associated with daily handling of snakes. It is also possible that safety or vicarious-extinction learning was acquired through the observation of learning models (other more experienced snake experts) [48, 49]. We also showed that indirect learning through socially acquired information may have similar immunizing effect against fear as was demonstrated for observant learning [30, 31]. That is, if people mostly encountered information that they felt influenced them not to fear snakes, they scored lower on measures of fear of snakes compared to those who reported encountering information that influenced them to fear snakes. This might have important practical significance in a clinical setting, using multiple approaches in therapy, combining information and exposure.

These results are partially in accordance with a recent study on the relation between experience with snakes and attitudes among Nigerian people [39]. The authors found that the more frequent encounters with snakes people had, the lower fear and disgust of snakes they reported. More frequent exposure to snakes was also associated with higher tolerance, which was consequently related to a reduced likelihood of intentional snake killing. However, in contrast to our data, a personal experience with snakebites had exactly the opposite effect as people who have been bitten by a snake themselves or knew someone from the community were also more likely to kill snakes. We may hypothesize that such negative influence of snakebite experience in their study might have been caused by high prevalence of fatal snakebites in that particular study area. There lives one of the most venomous snakes, the black-necked spitting cobra (*Naja nigricolis*), West African carpet viper (*Echis ocellatus*), and the western green mamba (*Dendroaspis viridis*). According to the authors, 31 participants (9.3%) reported being bitten by a snake, while 147 participants (43.9%) knew someone else who has received a snakebite. Moreover, 13 subjects (3.9%) had a family member killed by a snake and another 41 participants (12.2%) knew someone in their community who had suffered a fatal snakebite injury [39].

Our study is also in line that of Poulton and colleagues [50] showing that participants with lower fear were those who had previously sustained more injuries. The major role of experience in fear reduction [51–53] has been demonstrated for fear of dogs [34] or dental fears [35, 36] too. Thus, exposure seems to be able to suppress even the most prepared fears. It is also possible that humans are not prepared to learn fear, but to preferentially attend to certain stimuli [10, 18, 21] and snake’s idiosyncratic features tend to attract discriminating human attention preferentially. This might facilitate fear learning depending on both vicarious and direct experience.

Instead of merely addressing how much learning experience is necessary to acquire fear, it would be equally important to inquire how much experience is needed to learn not to fear particular stimuli (fear inoculation) and the potential consequences of lack of fear (hypophobia). Fear inoculation, similarly to preparedness, has its evolutionary significance. Inoculation can prove adaptive after gathering enough experience with the wildlife in a known territory and knowing which animals and individuals of a particular species are dangerous (see also [54]). The opposite approach, which is irrational, dysregulated fear, might lead to serious health issues [55] and impairments of cognitive functions [56, 57].

This work also brings about occupational, safety, and health implications that are worth mentioning. For example, Bawaskar and Bawaskar [58] noted that rural people in India inhabit sheds and mud houses without sanitation and with waste, tools, and firewood often close to their houses. This attracts rats and mice, which in turn encourage snakes to approach. As kraits move freely in and out of houses to hunt at night, people sleeping on the floor come into close contact with these snakes. Bawaskar and Bawaskar [58] observed that Muslims, however, irrespective of their poverty, always sleep on beds and that although numbers of Muslims and Hindus in the studied district (Mahad) were about the same, krait bites occurred only among Hindus. This is an excellent example of how awareness of the problem could, on its own, save many lives.

People who live closer to snakes are likely to be less afraid of them and are also more prone to become victims of snakebites due to enhanced exposure and lack of fear. For example, many snakebites occur not only among farmers that sleep on the floor [59, 60, but when farmers go to the toilet outside and do not wear shoes nor a lantern [61, 62]. Similarly, there are more victims of snakebites among people who keep snakes as pets [63–65], which is not surprising given their more frequent contact with serpents compared with other people.

### Limitations

Several limitations of our data need to be noted. Given the particular field of study, we had a limited access to snake experts, hence the lower sample size. Although we have calculated the statistical power for the planned tests to determine the required sample size, unfortunately, we could not incorporate different sample sizes into the power calculation. In future research, a larger sample size of people with experience interacting with snakes would be required to replicate these effects. Moreover, questions related to fear origins and etiology are based on retrospective self-reports, which may impair validity of the findings due to recall bias. A better grasp of this problem would require further studies collecting additional longitudinal data. It is also noteworthy that our questions conflated exposure to information related to the feared stimulus, and the influence this information on fear development. For example, someone who was exposed to snake-related information but did not develop a fear, and someone who was not exposed to snake-related information at all, might both provide the same answer on this scale. Finally, albeit the SNAQ-12 is a widely used and validated measure for fear of snakes with excellent discrimination power, it would be interesting to include a behavioral measure of fear, such as the Behavioral Avoidance Test (BAT) as it might lead to more reliable results.

## Conclusions

To conclude, our results provide new evidence that people who have more experience with snakes are less fearful of them, even after severe aversive experiences, i.e. snakebites. Furthermore, experience acquired through social transmission might also lessen fear of snakes. To fully understand the underlying mechanisms, future research should address how aversive and neutral personal experiences, as well as other social and cultural factors, could contribute to feeling in control and safe in the presence of a fearful object.

## Data Availability

The datasets generated and/or analyzed during the current study are not publicly available due to limitations of ethical approval involving the expert and firemen data and anonymity but are available from the corresponding author on reasonable request.

## Footnotes

1 A further analysis with being bitten or not as a grouping variable, experience as a covariant and SNAQ12 as the dependent variable revealed that people bitten by a snake scored lower than those who have never been bitten (F (1,105) = 6.53,*p* = 0.012, ɳ^2^_p_ = 0.06) while experience did not have a significant effect (F (1,105) = 1.30,*p* = 0.257).

2 In a separate analysis we also checked if there was an interaction between the grouping and covariant variable, but the effect was nonsignificant (F (1,104) = 0.91,*p* = 0.341).
